# A Case of Antineutrophil Cytoplasmic Antibody Vasculitis-Associated Acute Kidney Injury in a Patient With Asymptomatic COVID-19 Infection

**DOI:** 10.7759/cureus.35006

**Published:** 2023-02-15

**Authors:** Taruna Chandok, Rabih Nasr, Kalpana A Uday

**Affiliations:** 1 Internal Medicine, Bronx Care Health System, Bronx, USA; 2 Nephrology, Bronx Care Health System, Bronx, USA

**Keywords:** aki, vasculitis, pauci-immune crescentic glomerulonephritis, p-anca, covid 19

## Abstract

Vasculitis, or inflammation of blood vessels, is commonly seen with severe acute respiratory syndrome Coronavirus disease 2 (SARS-CoV-2). It is usually triggered by an autoimmune response induced by the virus, infection by the virus itself and trauma to the epithelial vessels caused by the release of cytokines. We present a case of antineutrophil cytoplasmic antibody (ANCA)-associated vasculitis (pauci-immune crescentic glomerulonephritis [GN]) superimposed on acute kidney injury caused by SARS-CoV-2. Our patient is a 57-year-old Hispanic female who presented with rising creatinine and active urinary sediment in the setting of an asymptomatic COVID-19 infection. A kidney biopsy was done for declining renal function, and positive myeloperoxidase antibodies revealed pauci-immune focal crescentic glomerulonephritis. Normalization of renal function was not achieved with pulse steroids and rituximab. The patient required long-term hemodialysis. Our case here adds to the very few cases of pauci-immune crescentic glomerulonephritis reported in patients with asymptomatic SARS-CoV-2 infection. We recommend keeping this high on the differential in SARS-CoV-2-infected patients presenting with acute kidney injury.

## Introduction

Antineutrophil cytoplasmic antibody (ANCA)-associated vasculitis (AAV) is immune complex-mediated damage to the small and medium-sized vessels. It is associated with ANCA specific for myeloperoxidase (MPO-ANCA) or proteinase 3 (PR3-ANCA). Though it can affect various organs, the lungs and kidneys are the most commonly affected. The term "renal-limited AAV" is used when it involves a single organ. AKI is the most common presentation. The incidence of acute kidney injury (AKI) in patients with Coronavirus-2019 (COVID-19) varies from 0.5% to 80% [1-3]. The severity of this condition determines the clinical outcome, prolongs the hospital stay, and increases the risk of the development of chronic kidney disease and end-stage renal disease in these patients. A multicenter retrospective cohort study involving critically ill patients with COVID-19 infection found a higher incidence of acute kidney injury and mortality in critically ill patients [4]. Various forms of acute kidney injury have been described in association with COVID-19, the most common being acute tubular injury and collapsing glomerulopathy. Immune complex glomerulonephritis (GN) is still a rare presentation in patients with active COVID-19 infection or post-COVID-19 infection. Rare cases of membranoproliferative GN affecting the kidney and microscopic polyangiitis manifesting as necrotizing glomerulonephritis and pulmonary capillaritis have also been reported with COVID-19 infection [5,6]. Age, sex, race/ethnicity, genetic predisposition, and environmental factors may influence its development. The data are still insufficient and need future research [7].

## Case presentation

A 57-year-old Hispanic female was sent from the clinic in January 2022 by her primary care physician for a creatinine level of 6.3 mg/dl with a baseline creatinine of 0.5 mg/dl in November 2021. The patient’s medical history was significant for well-controlled asthma, a history of childhood-treated tuberculosis, and a recent diagnosis of Mycobacterium intracellulare (MAI), for which she was started on rifabutin, azithromycin, and ethambutol on November 21. The patient reported no prior history or family history of connective tissue disorder, previous kidney injury or kidney disease, no alcohol or illicit drug use, or no recent travel. The patient was immunized with two doses of the COVID vaccine in June 2021 and September 2021. On arrival to the emergency department, the patient was asymptomatic, saturating well on room air, with no significant findings on physical examination.

Laboratory investigations at the time of presentation showed severe acute respiratory syndrome Coronavirus disease 2 (SARS-COV-2) RNA-positive, leukocyte count of 10.0k/µl (normal range 4.8-10.8 k/µl), eosinophil count of 0.20 k/µl (normal range 0.05-0.25 k/µl), hematocrit of 28.4% (normal range 42-51%), platelet count of 388 k/µl (normal range 150-400%), sodium of 139 mEq/l (normal range 135-145 mEq/l), potassium of 5.1 mEq/l (normal range 3.5-5 mEq/l), bicarbonate of 21 mEq/l (normal range 24-30 mEq/l), blood urea nitrogen of 67 mg/dl (normal range 6-20 mg/dl), creatinine of 6.3 mg/dl (normal range 0.5-1.5 mg/dl), and glomerular filtration rate of 7.29 ml/min/1.73 m2. Urine analysis showed a turbid appearance of urine with a specific gravity of 1.013 and a pH of 5.50, along with many red blood cells, few hyaline casts, a leucocyte count, epithelial cells, and no eosinophils. Urine albumin/creatinine ratio was 1479 mg/g with urine sodium of 74 mEq/l, urine creatinine of 103 mg/dl, Fena was 3.3%. A urine drug screen was negative.

Ultrasound of the kidneys showed increased echogenicity of the bilateral renal parenchyma, compatible with medical kidney disease, but no hydronephrosis, renal calculi, or perinephric focal fluid collection. CT scans of the abdomen and pelvis without contrast showed no evidence of hydronephrosis, calculi, or perinephric inflammatory changes.

Our patient had a recent admission when she underwent a chest CT with contrast. This resulted in mild form of acute kidney injury raising the serum creatinine to 1.5 mg/dl from her baseline creatinine of 0.5 mg/dl. Nephrology was consulted, and the patient was further evaluated for the causes of acute kidney injury. The differentials at this time are contrast-induced kidney injury, acute glomerulonephritis, or interstitial nephritis. The patient’s treatment for MAI (azithromycin, ethambutol, and rifabutin) was held in the setting of rising creatinine levels at the time of admission. Patient also had increased inflammatory markers with erythrocyte sedimentation rate of 51 mm/hr, c-reactive protein of 7.28 mg/l, D-dimer of 5503, underwent ventilation/perfusion scan which demonstrated multiple segmental, sub-segmental and non-segmental defects matching chest X-ray abnormalities, indeterminate for pulmonary embolism (PE). Patient was started on apixaban for suspected PE.

Creatinine started trending down from 6.3 mg/dl on admission to 5 mg/dl on day 5 of admission with the administration of normal saline. The patient was still saturating well on room air and was in no apparent distress with COVID pneumonia, now receiving antibiotics - ceftriaxone and doxycycline. Meanwhile the work up for acute glomerulonephritis; anti-nuclear antibodies (ANA) was negative, anti DNA antibodies were 2 IU/ml (< or = 4 IU/ml negative), serum C3 complement was 122 mg/dl (normal range 90-150 mg/dl), serum C4 complement was 33 mg/dl (normal range 16-47 mg/dl), serum immunoglobulin M level was 54 mg/dl (normal range 50-100 mg/dl), serum immunoglobulin A level was 310 mg/dl (normal range 47-310 mg/dl), immunoglobulin G level was1009 mg/dl (normal range 600-1640 mg/dl), glomerular basement membrane antibody was <1.0 AI indicating no antibody, hepatitis C antibody was negative, hepatitis B antigen and antibody were negative, Sjogren’s antibody (SS-A and SS-B antibody) was <1 AI indicating no antibodies. Work up for ANCA vasculitis - myeloperoxidase was 51.8 AI, proteinase-3 antibody was negative, raising the suspicion for pauci-immune glomerulonephritis/ANCA associated vasculitis. Rheumatology was consulted, and the patient was started on a pulse dose of 1000 mg methylprednisolone for three days followed by 60 mg of prednisone daily for an initial suspicion of rapid progressive glomerulonephritis. While awaiting a kidney biopsy, the patient was noted to have worsening of kidney function with the rise in creatinine from 5 mg/dl on day 5 to 6.7 mg/dl on day 12 of admission. The patient had no symptoms of uremia. Patient was re-tested for SARS-COV-2 RNA which was negative on day 14 with no significant improvement in kidney function (creatinine - 6.6mg/dl). A decision was made to provide acute hemodialysis in view of worsening kidney function and to plan a kidney biopsy to improve platelet dysfunction. The patient was explained in detail and consent obtained. On days 15 and 16, as well as the day before the scheduled kidney biopsy, the patient received three dialysis sessions.

**Table 1:** Creatinine trend per day of admission

Day	1	2	3	4	5	11	12	14	19	25	32
eGFR (ml/min)	7.29	7.97	7.71	8.21	9.51	8	8.52	6.91	13.46	15.15	9.09
Sodium (mEq/l)	139	144	138	145	147	145	144	141	142	146	139
Potassium (mEq/l)	5.1	4.8	4.4	4.6	4.9	4.8	5.2	5.0	3.8	3.4	4.6
Bicarbonate (mEq/l)	21	20	22	20	22	28	27	25	31	29	32
Blood urea nitrogen (mg/dl)	67	57	67	63	58	72	73	108	43	24	38
Creatinine (mg/dl)	6.3	5.8	6	5.7	5	5.8	6.7	6.6	3.7	3.3	5.2

**Figure 1:**
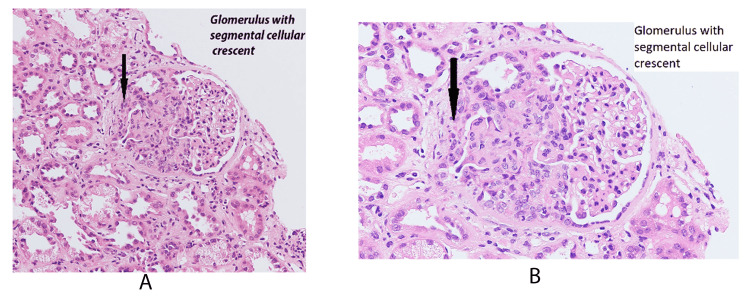
(A) Glomerulus with segmental cellular crescent; (B) zoom in of the glomerulus with segmental cellular crescent.

**Figure 2:**
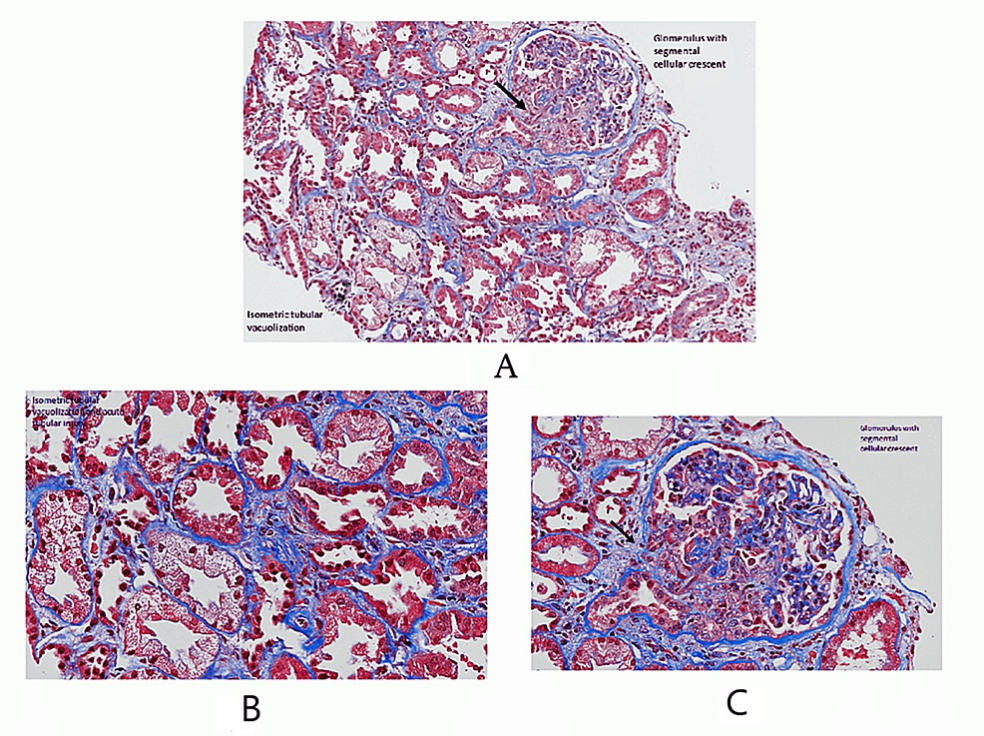
(A) Isometric tubular vacuolization and glomerulus with segmental cellular crescent; (B) isometric tubular vacuolization; and (C) glomerulus with segmental cellular crescent.

**Figure 3:**
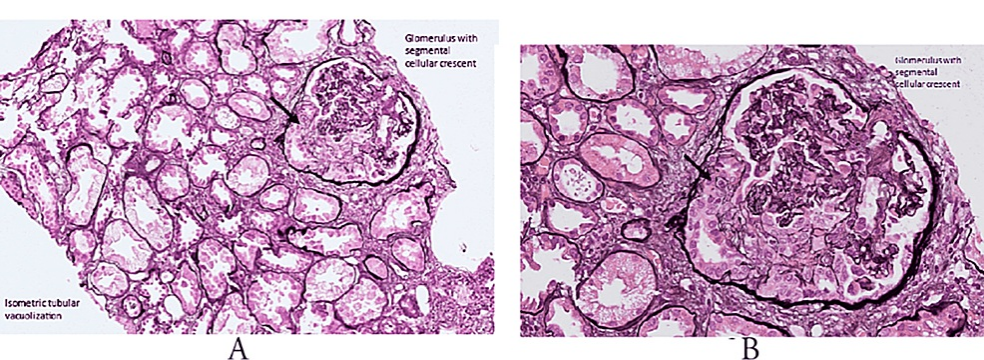
(A) Glomerulus with segmental cellular crescent and isometric tubular vacuolization; (B) zoom in glomerulus with segmental cellular crescent. Courtesy: Dr. Dominick Santoriello, MD; Dr. Geetha Jagannathan, MD; New York Presbyterian Hospital-Columbia University Medical Center-Anatomic Pathology Labs.

The patient successfully underwent a kidney biopsy with no complications. The results showed acute pauci-immune type, focal crescentic glomerulonephritis (MPO-ANCA positivity associated), and diffuse acute tubular injury with isometric tubular vacuolization (Figures 1A, 1B, 2A, 2B, 3A, 3B).

Rheumatology was re-consulted. The patient received one dose of rituximab, with a second dose planned two weeks later. The steroids were tapered. Her creatinine and glomerular filtration rate (eGFR) showed some improvement initially, a few days after rituximab and pulse dose steroids (creatinine: 3.5 mg/dl and eGFR: 14 ml/min). Creatinine started trending up to 5.2 mg/dl with an eGFR of 9.09 ml/min a week after Rituximab. Because a 24-hour urine collection showed creatinine clearance of 10 ml/minute in a volume of 850 ml and her kidneys did not show any signs of recovery post immunosuppressive therapy with rituximab, a decision was made to continue long-term hemodialysis. The patient was referred to an outpatient dialysis center with follow-up nephrology and rheumatology appointments.

## Discussion

With rising COVID-19 infections worldwide, the incidence of AKI is on the rise in these patients. It is one of the determinants of severity and fatality in COVID-19 patients [2]. Its incidence is 36.6% in patients hospitalized with COVID-19. Although various forms of AKI have been reported with COVID-19, ANCA-associated vasculitis (AAV) is still a rare presentation. Very few cases have been reported, most of them with both lung and kidney involvement [5]. Very few and rare cases of immune-mediated kidney injury have also been reported after the m-RNA COVID-19 vaccination [8,9]. These patients present with ground glass opacities, alveolar hemorrhage, and/or bilateral cavitary lesions. All these cases have been critically ill, requiring intensive care unit admission [10-12]. We here are presenting a case of immune-mediated kidney injury in an asymptomatic patient who presented with rising creatinine levels. Though there have been reported cases of viral and bacterial infections triggering autoimmunity and autoimmune diseases, it is interesting to note that SARS-CoV-2 infection can be a trigger factor for autoimmunity [13,14].

The pathophysiology involves the binding of SARS-CoV-2 to the angiotensin-converting enzyme 2 (ACE2) receptors, which explains its multi-organ involvement. These receptors are present on the arterial walls and on the endothelial cells of the veins. SARS-CoV-2 infection-induced autoimmunity involves an interplay of genetic, hormonal, immunological, and environmental factors. Molecular mimicry has been implicated in the development of various rheumatological diseases, including rheumatoid arthritis (RA), systemic lupus erythematosus (SLE), systemic sclerosis, and Sjogren syndrome [15,16]. Bystander activation and damage, persistent immune activation, and netosis are some of the other mechanisms inducing autoimmunity. The bystander damage starts with the migration of CD8+ T cells to the infected tissues. This leads to cell death and activation of the surrounding macrophages to release reactive oxygen species and nitric oxide, resulting in bystander killing of the surrounding cells. The presence of viral antigens is one of the driving mechanisms for immune proliferation. Ineffective clearance or persistence of SARS-CoV-2 cannot be ruled out as one of the mechanisms of autoimmunity. Netosis, the formation of neutrophil extracellular traps (NETs), is a phenomenon involved in trapping microorganisms to resolve the infection. Its formation was demonstrated by Zuo et al. by elevated serum levels of cell-free DNA, myeloperoxidase-DNA complexes, and citrullinated histone H3 among patients with COVID-19 [17].

The diagnosis of suspected renal-limited AAV involves a comprehensive history and physical examination, identifying recent drug exposures to rule out drug-induced AAV [18].

Laboratory testing includes testing for NACA, measuring serum creatinine, urine analysis with an examination of the urinary sediment, a complete blood count, measuring anti-nuclear antibody, anti-GBM antibody, C3 and C4 complement, cryoglobulins, testing for hepatitis B, C, and HIV, liver function tests, testing the patient for tuberculosis, blood cultures, and a CT chest imaging study. If the ANCA test is positive, a tissue biopsy of the affected organ confirms the diagnosis.

Treatment involves an induction phase and a maintenance phase. The induction therapy involves a rituximab- or cyclophosphamide-based regimen. Glucocorticoids in combination with rituximab or cyclophosphamide, rather than glucocorticoid monotherapy, are recommended. Glucocorticoid therapy is usually started with oral prednisone at a dose of 1 mg/kg per day, with a maximum dose of 60-80 mg/day. High-dose IV (pulse) glucocorticoids such as methylprednisone are reserved for patients presenting with rapidly progressive glomerulonephritis, such as our patient. AAV patients can also benefit from plasma exchange. It is based on the recommendations of the American College of Rheumatology/Vasculitis Foundation [19] and KDIGO [20]. Once remission is achieved after induction of immunosuppressive therapy, maintenance is achieved by treatment with rituximab, azathioprine, mycophenolate, or methotrexate. This is given for 12 to 24 months after the remission.

Our patient’s worsening renal function despite induction with immunosuppressive agents and steroids led to her re-admission a week later after discharge and the advancement of kidney injury to an end-stage renal disease requiring dialysis.

## Conclusions

Renal manifestations of COVID-19 have been well established, including acute kidney injury and various forms of vasculitis. Although AKI appears to be a marker of COVID-19 infection severity and mortality, early recognition and treatment can help prevent the progression of chronic kidney disease and end-stage renal disease. This case signifies that even a milder or asymptomatic COVID-19 infection can induce ANCA-associated vasculitis in patients with acute renal failure. Treatment with immunosuppressive agents and dialysis improves morbidity and mortality in such patients.

## References

[1] Ng JH, Bijol V, Sparks MA, Sise ME, Izzedine H, Jhaveri KD (2020). Pathophysiology and pathology of acute kidney injury in patients with COVID-19. Adv Chronic Kidney Dis.

[2] Shao M, Li X, Liu F, Tian T, Luo J, Yang Y (2020). Acute kidney injury is associated with severe infection and fatality in patients with COVID-19: a systematic review and meta-analysis of 40 studies and 24,527 patients. Pharmacol Res.

[3] Shetty AA, Tawhari I, Safar-Boueri L (2021). COVID-19-associated glomerular disease. J Am Soc Nephrol.

[4] Xu J, Xie J, Du B, Tong Z, Qiu H, Bagshaw SM (2021). Clinical characteristics and outcomes of patients with severe COVID-19 induced acute kidney injury. J Intensive Care Med.

[5] Allena N, Patel J, Nader G, Patel M, Medvedovsky B (2021). A rare case of SARS-CoV-2-induced microscopic polyangiitis. Cureus.

[6] Sethi S, D'Costa MR, Hermann SM, Nasr SH, Fervenza FC (2021). Immune-complex glomerulonephritis after COVID-19 infection. Kidney Int Rep.

[7] O'Shaughnessy MM, Hogan SL, Thompson BD, Coppo R, Fogo AB, Jennette JC (2018). Glomerular disease frequencies by race, sex and region: results from the International Kidney Biopsy Survey. Nephrol Dial Transplant.

[8] Klomjit N, Alexander MP, Fervenza FC (2021). COVID-19 vaccination and glomerulonephritis. Kidney Int Rep.

[9] Hakroush S, Tampe B (2021). Case report: ANCA-associated vasculitis presenting with rhabdomyolysis and pauci-immune crescentic glomerulonephritis after Pfizer-BioNTech COVID-19 mRNA vaccination. Front Immunol.

[10] Uppal NN, Kello N, Shah HH (2020). De novo ANCA-associated vasculitis with glomerulonephritis in COVID-19. Kidney Int Rep.

[11] Moeinzadeh F, Dezfouli M, Naimi A, Shahidi S, Moradi H (2020). Newly diagnosed glomerulonephritis during COVID-19 infection undergoing immunosuppression therapy, a case report. Iran J Kidney Dis.

[12] Jalalzadeh M, Valencia-Manrique JC, Boma N, Chaudhari A, Chaudhari S (2021). Antineutrophil cytoplasmic antibody-associated glomerulonephritis in a case of scleroderma after recent diagnosis with COVID-19. Cureus.

[13] Gracia-Ramos AE, Saavedra-Salinas MÁ (2021). Can the SARS-CoV-2 infection trigger systemic lupus erythematosus? A case-based review. Rheumatol Int.

[14] Zhou Y, Han T, Chen J (2020). Clinical and autoimmune characteristics of severe and critical cases of COVID-19. Clin Transl Sci.

[15] Cusick MF, Libbey JE, Fujinami RS (2012). Molecular mimicry as a mechanism of autoimmune disease. Clin Rev Allergy Immunol.

[16] Shah S, Danda D, Kavadichanda C, Das S, Adarsh MB, Negi VS (2020). Autoimmune and rheumatic musculoskeletal diseases as a consequence of SARS-CoV-2 infection and its treatment. Rheumatol Int.

[17] Zuo Y, Yalavarthi S, Shi H (2020). Neutrophil extracellular traps in COVID-19. JCI Insight.

[18] Weng CH, Liu ZC (2019). Drug-induced anti-neutrophil cytoplasmic antibody-associated vasculitis. Chin Med J (Engl).

[19] Chung SA, Langford CA, Maz M (2021). 2021 American College of Rheumatology/vasculitis Foundation guideline for the management of antineutrophil cytoplasmic antibody-associated vasculitis. Arthritis Rheumatol.

[20] Rovin BH, Adler SG, Barratt J (2021). Executive summary of the KDIGO 2021 Guideline for the management of glomerular diseases. Kidney Int.

